# Pivoting from polio eradication to measles and rubella elimination: a transition that makes sense both for children and immunization program improvement

**DOI:** 10.11604/pamj.supp.2017.27.3.11371

**Published:** 2017-06-21

**Authors:** Stephen L. Cochi

**Affiliations:** 1 Global Immunization Division, Centers for Disease Control and Prevention, Global Immunization Division, Atlanta, GA, USA

**Keywords:** Rubella, eliminating measles, immunization program

## Commentary

Why not take advantage of the experience and infrastructure developed to eradicate polio to pursue the elimination of measles and rubella, which is within our grasp [1, 2] ? The importance of eliminating measles, rubella and congenital rubella syndrome (CRS) is now recognized globally. Measles is still a leading cause of child deaths worldwide (>130,000 deaths per year) despite availability of an effective and cheap vaccine for more than 50 years [3]. Given the visibility of birth defects caused by Zika virus, we need to remember that rubella virus is still the leading infectious disease cause of congenital birth defects (CRS) globally, and the unfinished agenda of preventing through rubella vaccination the >100,000 CRS cases per year still occurring worldwide [4, 5]. So the measles and rubella vaccines continue to be “weapons of mass salvation” against these potentially deadly diseases.

With the establishment of a regional measles elimination goal in the African Region of WHO in 2011 and in the Southeast Asia Region in 2013, all six WHO regions have goals to eliminate measles by no later than 2020; the Global Vaccine Action Plan (GVAP) has a target for five of six WHO regions to achieve measles and rubella elimination by 2020 [6]. The Americas has already been declared free of endemic rubella in 2015 and endemic measles in 2016. However, for a variety of reasons including insufficient political commitment and resources, global progress toward reducing measles incidence and mortality has slowed during the past several years, making achievement of these targets on time unlikely. A recent external global review of the status of measles and rubella elimination, endorsed by WHO’s Strategic Advisory Group of Experts on immunization (SAGE), concluded that tremendous progress has been made toward both measles and rubella elimination since 2001 including significant gains during the period 2012-2015, but the initiative is not on track to achieve its ambitious goals by 2020 [7,8]. The review team stressed that “the basic strategies are sound; however, full implementation of these has been limited by lack of country ownership and global political will, reflected in insufficient resources.”

In 2011 both the WHO Executive Board and World Health Assembly declared that measles can and should be eradicated, with a target date to be determined. Both measles and rubella viruses are on the short list of candidates considered feasible and worthy of global eradication, together with the clear advantages, synergies, and cost effectiveness of pursuing their eradication simultaneously [1]. Disease eradication is the ultimate step in achieving global equity, and in this context can be pursued hand-in-hand with advancing achievement of the UN 2030 Sustainable Development Goal to end preventable deaths of newborns and children under 5 years of age by increasing vaccination coverage to levels needed to end vaccine preventable disease (VPD) deaths in children [9].

During nearly 30 years of operations, the Global Polio Eradication Initiative (GPEI) has mobilized and trained millions of volunteers, social mobilizers, and health workers; accessed households untouched by other health initiatives; mapped and brought health interventions to chronically neglected communities; and established a standardized, real-time global surveillance and response capacity [10,11]. Many of these polio assets have been applied in tandem to measles elimination, which has similar strategies and program implementation infrastructure needs. Since the beginning of GPEI in 1988, more than 15 million paralytic polio cases have been prevented through the use of polio vaccine; since 2000, 20.3 million measles deaths among children have been prevented with measles vaccination [3].

Does it make sense to wind down the polio assets and infrastructure during the next few years because of lack of forward vision and transition planning rather than pivoting to measles and rubella elimination in conjunction with activities to strengthen the overall immunization programs of countries? Having to reassemble this infrastructure later at greater expense and after lost momentum and human resource capacity would be challenging and inefficient. Moreover, the current half-hearted approach to pursuing measles-rubella elimination has later global consequences by gradually allowing children neither vaccinated nor exposed to these viruses to survive to older ages and even adulthood still susceptible, making the task of stopping transmission ever more complex, expensive, and difficult to achieve over the longer term. It is unacceptable not to seize the opportunity now to prevent the 134,200 annual measles deaths still occurring worldwide-61,600 (46%) of which are occurring in Africa (3)-together with the ongoing toll of 105,000 infants per year born with CRS-39,000 (37%) of which are in Africa (4). We need to build on experience and infrastructure accumulated to achieve the impending eradication of polio to also create a world free of both measles and rubella.

As the GPEI nears completion, the primary goals of transition planning for the initiative are both to protect a polio-free world and to ensure that these investments - made to eradicate polio - contribute to future health goals such as measles-rubella elimination and ending VPD deaths in children after the completion of polio eradication. These goals need to be closely coupled with enhanced efforts to strengthen overall immunization program performance needed to achieve GVAP national and subnational vaccination coverage targets, especially in the most vulnerable, low performing African countries [12,13]. Measles and rubella elimination is a perfect case study of how to achieve mutually reinforcing synergy with improving immunization program performance. Although there are challenges, building this synergy provides opportunities to draw on the abundant experience and lessons learned about approaches that work effectively [13]. A lesson learned from the polio eradication experience is the need to do a better job of closely linking measles-rubella activities with overall improvement of immunization program performance.

The following tactical approaches can be used to link strategies for measles-rubella elimination with strategies for immunization program strengthening: 1) Using measles outbreaks to visibly signal areas where immunization service delivery is less than optimal, and to drive prioritization of targeted interventions to improve program performance and ensure accountability [12]. Measles is an ideal program performance indicator (compared with, for example, polio) because of its frequency, contagious potential, and visibility since virtually every case is clinically apparent with rash and fever. Using measles disease incidence as an outcome measure of real disease burden, rather than only using vaccination coverage as a process measure, can help immunization programs identify and focus on populations at risk regardless of age. In addition, focused efforts to strengthen measles-rubella surveillance can be used to strengthen surveillance for other outbreak-prone vaccine-preventable diseases. 2) Using the introduction of a second dose of measles-containing vaccines (MCV2) to create new opportunities to receive vaccines and other child health interventions in the second year of life and beyond. In addition to administration of MCV2, a second year of life vaccination platform can provide an opportunity to catch up on vaccines missed during the first year, improve coverage with diphtheria-tetanus-pertussis (DTP) boosters needed beyond the first year of life, and provide a foundation for the introduction of new vaccines anticipated to have scheduled doses during the second year of life (e.g., meningococcal A conjugate vaccine, malaria vaccine). An additional well child visit during the second year of life also creates an opportunity to integrate immunization services with other health interventions, such as vitamin A supplementation and presumptive treatment for intestinal helminths. 3) Using measles-rubella vaccination campaign planning, training and implementation to identify and target chronically underserved populations and geographies, and to enhance the capability of routine immunization service delivery to equitably reach children. 4) Using advocacy for measles elimination to support institutions and policies needed for sustainable high quality immunization programs. Measles outbreaks are much more effective than low vaccination coverage in gaining the attention of political leaders and building the political will needed to increase investments in immunization programs, including institutional capacity in ministries of health and national public health institutes; workforce capacity, performance and accountability; and sustainable immunization financing.

As a practitioner who has spent decades on polio eradication and managing immunization program activities - and as an advocate for both measles-rubella elimination and strong immunization programs - my view is that transitioning from eradicating polio to measles-rubella elimination and immunization program strengthening is a no-brainer. It is both an opportunity and an obligation that should be taken for compelling reasons, including the close relationship between these two initiatives.

Here are a few reasons why this makes sense and how the polio infrastructure can be - and already is - readily harnessed for measles and rubella elimination while building overall immunization program capacity: First, the strategies used to eradicate polio are very similar to those for measles-rubella elimination. These strategies include disease detection and use of a laboratory network for diagnostic confirmation coupled with strong outbreak preparedness and response; the importance of achieving and maintaining high national and subnational vaccination coverage; and the need for periodic high quality supplementary immunization campaigns to reach children who lack access to routine immunization services. Second, the infrastructure required to eradicate polio and that will be needed to successfully eliminate measles and rubella is concentrated in many of the lowest-performing countries, which are the most challenging places to achieve any health objectives. Now is the time to determine how this massive infrastructure created for polio eradication can be sustained and repurposed for measles-rubella elimination and structured in a way that comprehensively improves immunization programs. Third, transitioning the polio assets for measles-rubella elimination and building overall immunization program capacity will sustain and extend the side benefits these resources have already provided, while at the same time maintaining and mainstreaming essential polio functions - such as disease detection, outbreak preparedness and response, immunization service delivery, and communications and community engagement -which will continue to be needed in immunization programs after polio eradication is certified worldwide.

GPEI has documented many important lessons learned that must be harnessed and applied to measles-rubella elimination, in conjunction with strengthening national immunization programs [10,11]. Here are some of the essentials we cannot afford to lose: 1) Knowledge and best practices accumulated on communications and community engagement, mobilizing social and community support for vaccination, and using a targeted disease elimination initiative like measles or polio eradication as a springboard for broader health communication. These lessons and experience have been generated in the most challenging countries in the world including India, Nigeria, Pakistan, and Afghanistan. 2) The value of an advanced, state-of-the-art global, regional and national laboratory network and real-time disease detection and response. I have seen in many countries the knowledge and resources of networks developed and supported for polio detection and response applied effectively to measles and other vaccine preventable diseases. In Nigeria, the use of the polio-funded human resources, infrastructure, and experience with the polio emergency operations center was instrumental in stopping Ebola virus transmission in its tracks after only 20 cases [14]. 3) The knowledge and experience acquired on how to reach every child, including the most underserved, migrants, nomads, people living in conflict zones and others marginalized by circumstances that prevent or impede access to health services. 4) Many examples of outstanding program monitoring and the use of accountability frameworks to assess performance in polio eradication, including in difficult settings such as Nigeria and Pakistan. 5) Partnership coordination, advocacy and resource mobilization were essential to achieving polio eradication goals. GPEI has assembled an unprecedented and committed global partnership led by Rotary International, World Health Organization, UNICEF, Centers for Disease Control and Prevention, and the Bill & Melinda Gates Foundation, which has collectively and relentlessly worked together to overcome the many challenges the initiative has faced, and whose vanguard is the 20 million frontline vaccinators. This largest-ever global health partnership is in an ideal strategic position to move forward on other global health challenges, such as the effort to wipe the measles and rubella viruses off the face of the earth and end VPD deaths among children.

Transitioning from polio to measles-rubella elimination and immunization program strengthening will not be easy, requiring agility, good planning, and strong leadership and management. We are already experiencing many of the growing pains and challenges associated with the task, such as complacency of countries and partner organizations in addressing off-track GVAP targets (6). We must guard against the compartmentalization of polio-measles-routine immunization staff and programs into separate silos of work and at times, competing interest groups. Even though it is hard work to transition, disease elimination programs are not a zero-sum game - successfully repurposing resources and knowledge from polio eradication to measles-rubella elimination is a win-win, especially for the world’s children. In short, the end of polio will not be only an incredible achievement in itself, but will open the door to protect the vulnerable from numerous diseases such as measles and rubella that kill and injure children.

Figure 1

### So what are we waiting for?

If we do not thoroughly plan and implement actions now to ensure that the legacy of polio eradication is optimized, the only losers will be the world’s children. For me, as the next step forward, measles-rubella elimination pursued in close conjunction with improving immunization program capacity needed to equitably increase vaccination coverage to levels needed to end VPD deaths in children is a no-brainer.

**Figure 1:**
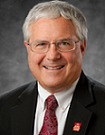
Steve Cochi

## Competing interests

The author declare no competing interest.
